# Placental serotonergic system dysregulation is associated with early impairments in infant neurodevelopment

**DOI:** 10.3389/fendo.2025.1694018

**Published:** 2025-12-19

**Authors:** Arturo A. Canul-Euan, Arturo Flores-Pliego, Juan M. Solis-Paredes, Aurora Espejel-Nuñez, Sandra B. Parra-Hernández, Tahiri Mendoza-Hernández, María del Carmen Hernandez-Chavez, Gabriela Gil-Martínez, Carmen J. Zamora-Sánchez, Otilia Perichart-Perera, Guadalupe Estrada-Gutierrez, Ignacio Camacho-Arroyo

**Affiliations:** 1 Star Medica, Hospital Infantil Privado, Mexico City, Mexico; 2 Centro de Estudios Avanzados sobre Violencia - Prevención (CEAVI-P), Instituto Nacional de Pediatría, Mexico City, Mexico; 3 Department of Immunobiochemistry, Instituto Nacional de Perinatología, Mexico City, Mexico; 4 Department of Reproductive and Perinatal Health Research, Instituto Nacional de Perinatología, Mexico City, Mexico; 5 Department of Developmental Neurobiology, The National Institute of Perinatology Isidro Espinosa de los Reyes, Mexico City, Mexico; 6 Nutrition and Bioprogramming Coordination, Instituto Nacional de Perinatología, Mexico City, Mexico; 7 Unidad de Investigación en Reproducción Humana, Instituto Nacional de Perinatología-Facultad de Química, Universidad Nacional Autónoma de México, Mexico City, Mexico

**Keywords:** neurodevelopment, serotonin, infant, humans, placenta

## Abstract

**Introduction:**

Serotonin (5-hydroxytryptamine [5-HT]) plays a fundamental role in fetal neurodevelopment. While 5-HT is synthesized in the fetal brain, the placenta contributes significantly to fetal 5-HT levels during early gestation. However, little is known about the influence of placental serotonergic components on early neurodevelopmental outcomes. In this study, we have evaluated the association between the expression of key components of the placental serotonergic system and neurodevelopmental status in infants of mother–child dyads enrolled in the Origen Bioquímico y Epigenético del Sobrepeso y la Obesidad (OBESO) perinatal cohort at the first month of life.

**Methods:**

We analyzed 5-HT concentrations in maternal serum, umbilical cord serum, and placental tissue, and investigated the expression of key proteins of the serotonergic system in the placenta. All samples were obtained from full-term healthy pregnancies. 5-HT levels were measured by ELISA, and protein expression in placental tissue was evaluated by Western blot. Neurodevelopment was assessed at 1 month of age using the Bayley Infant Development Scale III (BSID-III). Infants with two or more BSID-III domain scores ≤ 7 were grouped as having altereded neurodevelopment.

**Results:**

Placentas from infants with altered neurodevelopment exhibited higher expression of tryptophan hydroxylase types 1 and 2 (TPH1 and TPH2)—the rate-limiting enzymes in 5-HT synthesis—as well as the serotonin transporter (SERT), compared to those from infants with normal neurodevelopment. In contrast, expression of monoamine oxidase-A (MAO-A), the primary degrading enzyme, was significantly lower in the altered group. Interestingly, 5-HT levels and the expression of 5-HT1E and 5-HTR5A receptors were similar between groups.

**Conclusion:**

These findings suggest that dysregulation of the placental serotonergic system, independent of total 5-HT levels, could be associated with early neurodevelopmental impairments, highlighting the importance of placental serotonin signaling in fetal brain development.

## 1 Introduction

Neurodevelopment is a highly regulated and complex process that begins in early gestation and continues beyond birth. During this critical period, the development of the central nervous system (CNS) is shaped by genetic programming as well as biochemical and environmental influences, which can lead to long-term deficits in motor, cognitive, language, and behavioral skills (1). Among the key intrinsic factors involved is serotonin, or 5-hydroxytryptamine (5-HT), a biogenic amine that regulates numerous physiological functions across various tissues, including the brain (2, 3). Major sources of 5-HT include enterochromaffin cells in the small intestine, raphe neurons in the brainstem, and the placenta.

Tryptophan hydroxylase (TPH), the rate-limiting enzyme in 5-HT synthesis, exists in two isoforms: TPH1, mainly expressed in the peripheral nervous system, and TPH2, predominant in the CNS. TPH hydroxylates l-tryptophan to produce l-5-hydroxytryptophan, which is then converted into 5-HT by aromatic amino acid decarboxylase. Once released, 5-HT is taken up by its transporter (serotonin transporter [SERT]). It exerts its effects through interaction with membrane-bound receptors (5-hydroxytryptamine receptors [5-HTRs]), which comprise seven main types (5-HT1 to 5-HT7) and 14 subtypes. Most 5-HTRs are G protein-coupled receptors, except for the cationic channel-dependent 5-HT3 subtype (4, 5). Finally, 5-HT is catabolized by monoamine oxidase A (MAO-A) into 5-hydroxyindoleacetic acid (5-HIAA).

Beyond its well-established role as a neurotransmitter, 5-HT is also involved in key developmental processes, participating in blastocyst implantation and placental development (2, 6). Elements of the serotonergic system have been identified in early embryonic stages in *Xenopus* (7), the human placenta (8), and amniotic epithelial cells, supporting its involvement in fetal development (9). Disruptions in placental 5-HT production have been linked to an increased risk of autism spectrum disorders (10, 11) and fetal growth restriction (12). Both excessive and deficient placental 5-HT synthesis and transport are implicated in fetal neurodevelopmental changes associated with ASD. Altered expression of TPH1/TPH2, MAO-A, and SERT, as well as certain TPH1 polymorphisms affecting its activity (13, 14), alters 5-HT levels, impacting fetal brain development early during gestation. In addition, 5-HT is actively synthesized by placental syncytiotrophoblasts by the 11th week of gestation in humans, and E10.5–E14.5 in rodents. In mouse models and humans, the relevance of placental 5-HT for brain development, even before the establishment of serotonergic brain innervation and the dorsal raphe nucleus (the main source of 5-HT for the fetal brain), has been demonstrated (15, 16). In mice, placental 5-HT synthesis by 5-TPH1 is essential for forebrain development (15). Beyond its role in placental function, 5-HT also promotes cell proliferation, migration, and neural arborization (15, 16).

Alterations in 5-HT levels during critical windows of brain development, such as gestation and the first year postpartum, may have lasting effects on cognitive functions, sensory processing, social behavior, and executive function (17), contributing to conditions such as depression, anxiety, and autism (3, 18). The placenta plays a key role in the synthesis, metabolism, and transport of 5-HT to the fetus, thereby influencing neurodevelopment (19). Epigenetic modifications of the placental *HTR2A* gene, which encodes the 5-HT2A receptor, have been associated with early neurobehavioral outcomes, such as movement quality and attention (19). Moreover, maternal stress during pregnancy has been linked to increased placental MAO-A expression, which in turn correlates with heightened negative reactivity and reduced emotional regulation in infants at 12 months of age (20). In this study, we assessed neurodevelopment at 1 month of age using the Bayley Infant Development Scale III (BSID-III), a standardized scale for infants from 1 to 42 months (21, 22). Developmental milestones observable at this age include responsive smiling, eye tracking of objects, reflexes such as grasp and startle, reaction to sounds, and vocalizations (21, 22). Assessing neurological development at 1 month of age may have limited sensitivity due to the inherent constraints of early assessment, which we minimized by applying the scale through trained and standardized personnel to ensure consistency, and by analyzing the data using both continuous and bivariate scores to strengthen interpretation of the results.

Understanding alterations in the maternal–placental serotonergic system and their impact on early neurodevelopment may offer valuable insights into the mechanisms underlying the developmental origins of mental health disorders. Therefore, this study aimed to investigate the expression of key serotonergic components in the human placenta and to explore their association with infant neurodevelopmental status at 1 month of age.

## 2 Materials and methods

### 2.1 Participants

This study included healthy pregnant Mexican women enrolled in the Origen Bioquímico y Epigenético del Sobrepeso y Obesidad (OBESO) perinatal cohort at the Instituto Nacional de Perinatología (INPer), Mexico City. Details of the cohort have been described previously (23).

All participants completed a structured questionnaire that included medical and obstetric history, demographic data, and self-reported pregestational weight. Inclusion criteria were singleton pregnancy, absence of obstetric complications, and no use of glucocorticoids, anxiolytics, or antidepressants during pregnancy. Height was measured using a stadiometer (SECA 264, Hamburg, Germany), and pregestational body mass index (pBMI) was calculated. Women were classified according to the World Health Organization criteria. Weight was measured during pregnancy using a calibrated digital scale (Tanita BWB-800, Tanita Corporation, Tokyo, Japan). Gestational weight gain (GWG) was defined as the difference between the weight at the last prenatal visit and the pregestational weight. It was categorized as insufficient, adequate, or excessive according to the Institute of Medicine’s 2009 guidelines. Systolic and diastolic blood pressure were measured with a sphygmomanometer using standardized procedures, ensuring participants were at rest. A 75-g oral glucose tolerance test was performed after an overnight fast of at least 8 h, with glucose measured at fasting, 1 h, and 2 h (24).

Maternal serum determinations of glucose, triglycerides, total cholesterol, high-density lipoprotein cholesterol (HDL-C), and low-density lipoprotein cholesterol (LDL-C) were measured using an automated analyzer (ISE Echo Lory 2000) and commercial kits (DiaSys Diagnostics Systems GmbH, Holzheim, Germany). All neonates were healthy and delivered at full term (37–39 weeks of gestation). Neonatal weight and length were measured using a pediatric scale (Tanita BWB-800, Tanita Corporation, Tokyo, Japan) and an infantometer (SECA 207, Hamburg, Germany). Sex and Apgar scores at 1 and 5 min were obtained from the clinical records. Neurodevelopmental assessment was performed at 1 month of age as part of the cohort follow-up.

### 2.2 Neurodevelopmental assessment

Infant neurodevelopment at 1 month of age was assessed using the BSID-III, which evaluates six domains: cognitive, receptive language, expressive language, fine motor, gross motor, and socioemotional. All evaluations were conducted at the research center by licensed infant psychologists, with each assessment lasting approximately 15–25 min. Mothers also completed the BSID-III socioemotional questionnaire. Scalar scores (mean = 10, SD = 3) were calculated for each domain, as per the test manual. Altered neurodevelopment was defined as a scalar score ≤ 7, that is, ≥ 1 SD below the mean in at least one domain (25, 26).

### 2.3 Sample collection

Infants were classified into normal or altered neurodevelopment groups based on BSID-III results, and corresponding placental samples were selected from the OBESO biobank (*n* = 49). Maternal blood was collected in serum separator tubes containing a clot activator tube prior to delivery, and cord blood was obtained immediately after birth. After allowing the samples to clot at room temperature for 30 min, they were centrifuged to obtain serum, which was then aliquoted and stored at − 70°C until analysis. Placental tissue, including both maternal and fetal sides, was collected within 10 min of delivery, transported under sterile, temperature-controlled conditions (4°C), and stored at − 70 °C until analysis.

Tissue samples were taken approximately 5 cm from the central region of the placental disc, near the umbilical cord insertion site, to ensure consistency among specimens. For both Western blot and 5-HT enzyme-linked immunosorbent assay (ELISA) analyses, fragments were obtained from this placental tissue area, which contained a representative mixture of maternal and fetal components. The same sampling procedure was uniformly applied to all cases to maintain methodological comparability among groups.

### 2.4 ELISA assay

5-HT levels were measured in maternal serum, umbilical cord serum, and placental tissue (*n* = 49) using an ELISA kit (Cat. No. KA1894; Abnova, Jhongli, Taoyuan, Taiwan) with a detection limit of 6.2 ng/mL. All samples were acetylated according to the manufacturer’s instructions and analyzed in duplicate. Concentrations were calculated using a four-parametric logistic regression standard curve. Optical density was measured at 450 nm with a microplate reader (Synergy HT, Biotek Instruments, Winooski, VT, USA).

### 2.5 Protein extraction

Placental tissue was rinsed with cold 1× PBS to remove erythrocytes. Approximately 40 mg of placental tissue was homogenized in 1 mL of RIPA buffer (Cat. No. 89900; Thermo Fisher Scientific, Waltham, MA, USA) supplemented with Halt^®^ 1× protease inhibitor (Cat. No. 87786; Thermo Fisher Scientific, Waltham, MA, USA). Homogenization was performed under cold conditions using an electric homogenizer to ensure efficient tissue disruption. The homogenates were subsequently passed through a 26-G needle to ensure complete cell lysis and then centrifuged at 14,000 rpm at 4°C for 20 min. Protein concentration was determined using the bicinchoninic acid (BCA) assay (Cat. No. 23227; Thermo Fisher Scientific, MA, USA) (27). Supernatants were collected and stored at − 70°C until use.

### 2.6 Western blot analysis

A subset of placentas was selected for Western blot analyses (*n* = 10; normal *n* = 5, altered *n* = 5) based on protein quality, quantity, and biological representativeness (those classified as altered presented two or more domains with scores ≤ 7). Equal amounts of protein (5 µg) were resolved on 10% SDS-PAGE and transferred to polyvinylidene fluoride membranes (Millipore, Billerica, MA, USA). Proteins of interest were detected with the following antibodies (28): rabbit monoclonal anti-TPH-1 (1:500, Cat. No. Ab52954; Abcam, Cambridge, MA, USA), polyclonal rabbit anti-TPH2 (1:100, Cat. No. NB100-7455; Novus Biologicals, Littleton, CO, USA), rabbit monoclonal anti-SERT (1:5,000, Cat. No. Ab181034; Abcam, Cambridge, MA, USA), rabbit monoclonal anti-MAO-A (1.5,000, Cat. No. Ab 126751; Abcam, Cambridge, MA, USA), rabbit polyclonal anti-5-HT1E (0.4 µg/mL, Cat. No. NBP1-90320: Novus Biologicals, Littleton, CO, USA), mouse monoclonal anti-5-HTR5A (1:500, Cat. No. A100198; Antibodies, St Louis, MO, USA), and, as internal control mouse, monoclonal anti-GAPDH (1:5,000 Cat. No. Ab8245, Abcam, Cambridge, MA, USA). Membranes were subsequently incubated with secondary antibodies: goat antirabbit IgG (1:5,000, Cat. No. Ab6721; Abcam, Cambridge, MA, USA) or rabbit antimouse IgG conjugated with horseradish peroxidase (1:2,000, Cat. No. Ab6728; Abcam, Cambridge, MA, USA). Proteins were detected using SuperSignal™ West Femto Maximum Sensitivity Substrate (Thermo Scientific, Waltham, MA, USA) chemiluminescence reagents and visualized on X-ray film. Densitometric analysis was performed with Fiji-ImageJ software (National Institute of Mental Health, Bethesda, MD, USA) (29).

### 2.7 Statistical analysis

Descriptive statistics and frequency analysis were used to describe the study population. Continuous variables were expressed as mean ± standard deviation (SD) or median and interquartile range (IQR). Data were analyzed using the Student’s t test or the Mann–Whitney *U* test, and categorical variables were analyzed with the Chi-square test. A multivariable binary logistic regression was performed to assess the association between maternal and neonatal variables and neurodevelopmental outcomes. Results are presented as odds ratios (OR) with 95% confidence intervals (95% CI). A stepwise multiple linear regression model was performed to determine which variables were associated with various domain scores of neurodevelopment. Statistical significance was considered at *p* < 0.05. All analyses were performed using GraphPad Prism 6.0 software (GraphPad, San Diego, CA, USA).

## 3 Results

### 3.1 Infant neurodevelopmental outcomes

A total of 49 mother–infant dyads were included in the study. Of these, 32.7% (*n* = 16) of infants demonstrated normal global neurodevelopment, while 67.3% (*n* = 33) exhibited alterations in at least one domain. All infants were born at term, with a median gestational age of 38.6 (SD = 1.13). No significant differences were observed in maternal characteristics between the normal and altered neurodevelopment groups.

Regarding neonatal characteristics, the Apgar score at 1 min was significantly lower in the altered group (*p* = 0.010). Additionally, there was a trend toward a higher proportion of male infants in the altered neurodevelopment group (*p* = 0.054). Maternal and neonatal characteristics by neurodevelopmental status are summarized in **Table 1**.

**Table 1:** Clinical and biochemical characteristics of mothers and their infants according to neurodevelopmental status.

Variables	All participants (*n* = 49)	Normal neurodevelopment (*n* = 16)	Altered neurodevelopment (*n* = 33)	*p*-value
Maternal characteristics				
Maternal age (years)	31.8 (4.09)	31.9 (3.51)	28.4 (7.17)	0.887
pBMI (kg/m^2^)	26.7 [23.8–30.3]	26.8 [23.8–29.4]	26.0 [23.6–32.0]	0.725
pBMI classification (*n*, %)				
Normal weight	20 (40.8)	6 (37.5)	14 (42.4)	0.615
Overweight	14 (28.6)	6 (37.5)	8 (24.2)	
Obesity	15 (30.6)	4 (25.0)	11 (33.3)	
GWG (kg)	6.53 (5.10)	7.66 (3.53)	5.98 (5.68)	0.284
GWG classification (*n*, %)				
Adequate	12 (24.5)	5 (31.3)	7 (21.2)	0.730
Insufficient	31 (63.3)	9 (56.3)	22 (66.7)	
Excessive	6 (12.2)	2 (12.5)	4 (12.1)	
Maternal comorbidity	None	None	None	
Primiparous (*n*, %)	25 (51.0)	10 (62.5)	15 (45.5)	0.263
Glucose (mg/dL)	77.4 (11.5)	80.8 (14.5)	75.7 (9.47)	0.147
Triglycerides (mg/dL)	194 (51.2)	199 (49.5)	191 (52.7)	0.663
Cholesterol (mg/dL)	243 (54.1)	233 (54.5)	249 (53.8)	0.331
HDL-C (mg/dL)	64.9 (17.3)	69.2 (18.9)	62.8 (16.3)	0.226
LDL-C (mg/dL)	128 (42.1)	11 (18.6)	133 (49.2)	0.111
SBP (mmHg)	100 [90.0–110]	103 [90.0–112]	100 [90.0–109]	0.326
DBP (mmHg)	60.0 [57.3–63.0]	60.0 [52.0–66.0]	60.0 [58.0–61.5]	0.872
OGTT-fasting (mg/dL)	79.5 (7.53)	82.9 (7.33)	78.0 (7.09)	0.052
OGTT-1 h (mg/dL)	133 (33.0)	140 (33.2)	130 (33.0)	0.382
OGTT-2 h (mg/dL)	106 (22.9)	102 (16.9)	108 (25.0)	0.444
Infant characteristics				
GA at birth (weeks)	38.6 (1.13)	38.5 (0.94)	38.5 (1.23)	0.792
Sex (*n*, %)				
Female (1)	24 (49.0)	11 (68.8)	13 (39.4)	0.054
Male (2)	25 (51.0)	5 (31.3)	20 (60.6)	
Born from cesarean section (*n*, %)	35 (71.4)	10 (62.5)	25 (75.8)	0.335
Apgar 1 min	8 [8–8] (6–9)	8 [8–9] (8–9)	8 [8–8] (6–9)	**0** **.010^a^**
Apgar 5 min	9 [9–9] (9–10)	9 [9–9] (9–10)	9 [9–9] (9–10)	1.00^a^
Birth weight at birth (g)	3,060 (0.43)	2,970 (0.35)	3,020 (0.46)	0.691
Birth height at birth (cm)	47.7 (2.08)	47.6 (1.75)	47.8 (2.23)	0.688
Head circumference at birth (cm)	33.7 (1.19)	33.7 (1.19)	33.7 (1.21)	0.979
Weight at 1 month (g)	4,040 (0.53)	4,040 (0.39)	3,980 (0.59)	0.711
Height at 1 month (cm)	52.5 (1.92)	52.3 (2.00)	52.5 (1.90)	0.654
Head circumference at 1 month (cm)	36.8 (1.22)	36.8 (1.31)	36.7 (1.20)	0.957

*pBMI*, pregestational body mass index; *GWG*, gestational weight gain; *GA*, gestational age; *HDL-C*, high-density lipoprotein cholesterol; *LDL-C*, low-density lipoprotein cholesterol; *SBP*, systolic blood pressure; *DBP*, diastolic blood pressure; *OGTT*, oral glucose tolerance test; *Apgar 1*, Apgar score at 1 min; *Apgar 5*, Apgar score at 5 min. Continuous variables are expressed as median [IQR] and mean (SD) and compared using the *t*-test or Mann–Whitney *U* test. Categorical variables are expressed as a number (percentage) and compared using the *χ*² test. Sex of newborn was coded as 1 = female and 2 = male. ^a^Mann–Whitney test with Monte Carlo permutation (two-tailed) and values are median [IQR] (min–max).

Variables that showed significant differences are indicated in bold.

**Figure 1:**
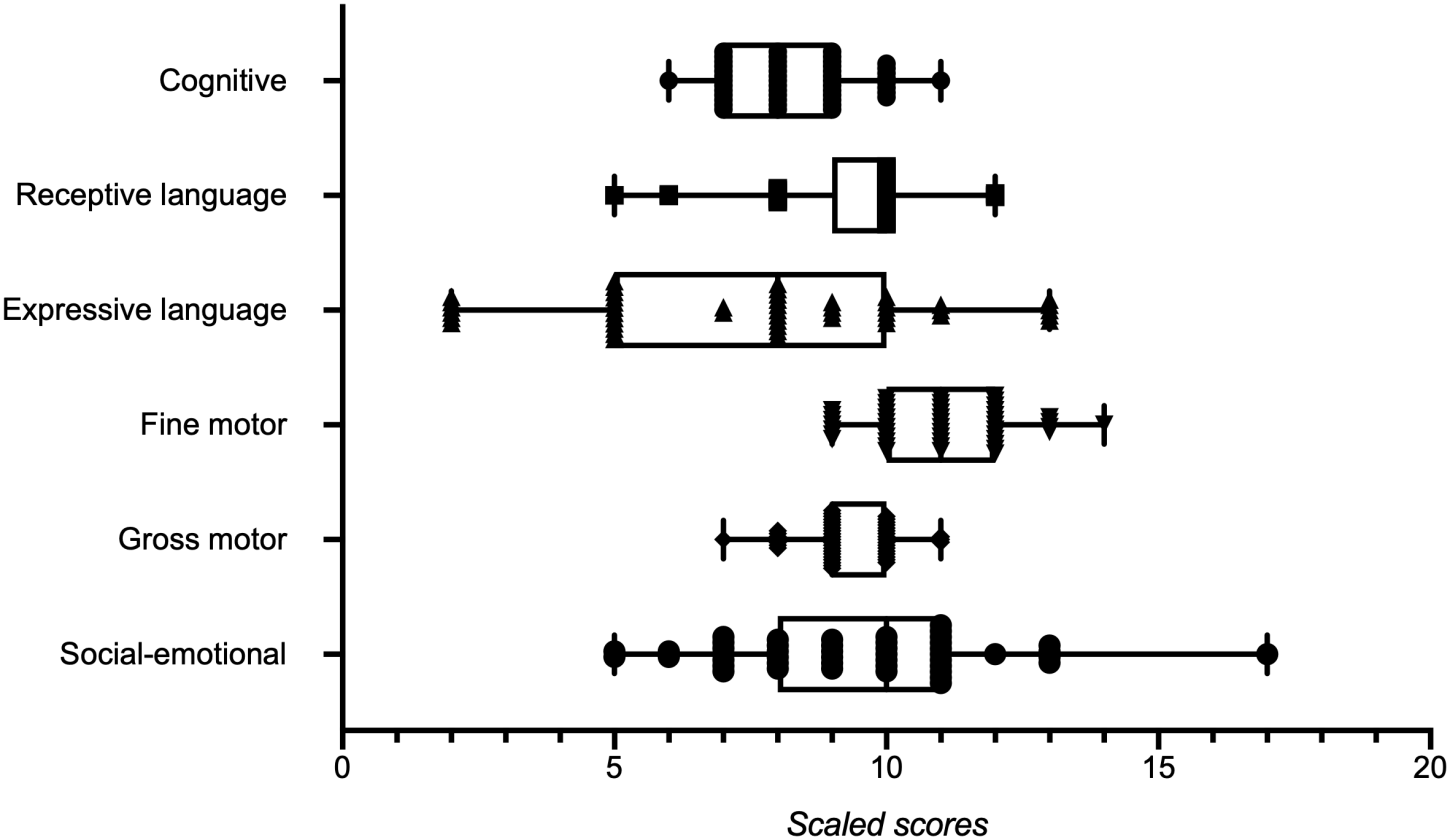
Scaled score for the different neurodevelopmental domains assessed using the BSID-III test. Box and whisker plot showing the maximum and minimum values. All points are displayed.

The binary logistic regression model identified a modest association between maternal cholesterol levels and neurodevelopment (*β* = 0.03, *p* = 0.045; OR = 1.03, 95% CI: 1.00–1.05). The complete results of the model are presented in **Table 2**. **Figure 1** shows the distribution of scaled scores for each domain of the BSID-III test. Multiple linear regression identifies associations between maternal and infant characteristics and scaled scores on the cognitive, fine motor, and socioemotional domains of the BSID-III test. The significant models are presented in **Table 3**.

**Table 2:** Results of binary logistic regression analysis for neurodevelopmental outcome in infants.

Variable	Beta	SE	*p*-value	OR	95% CI
Maternal age (years)	0.01	0.13	0.941	1.01	0.79–1.30
pBMI (kg/m^2^)	− 0.05	0.09	0.620	0.96	0.80–1.15
Parity	− 0.20	1.01	0.841	0.82	0.13–5.91
Glucose (mg/dL)	− 0.11	0.06	0.067	0.90	0.80–1.01
Triglycerides (mg/dL)	− 0.02	0.01	0.184	0.98	0.96–1.01
Cholesterol (mg/dL)	**0.03**	**0.01**	**0.045**	**1.03**	**1.00**–**1.05**
HDL-C (mg/dL)	− 0.08	0.05	0.088	0.93	0.85–1.01
LDL-C (mg/dL)	0.02	0.03	0.219	1.02	0.99–1.06
GWG (kg)	− 0.23	0.15	0.122	0.79	0.60–1.06
GA at birth (weeks)	0.09	0.51	0.857	1.10	0.41–2.97
Mode of delivery	− 1.06	1.65	0.520	0.35	0.01–8.75
Sex of newborn	− 1.92	1.50	0.200	0.15	0.01–2.76
Birth weight at birth (g)	0.004	0.004	0.221	1.00	0.99–1.01
Birth height at birth (cm)	− 0.38	0.43	0.376	0.68	0.29–1.59
Head circumference at birth (cm)	− 0.89	0.83	0.285	0.41	0.08–2.09
Constant	44.6	36.0	0.216		

*pBMI*, pregestational body mass index; *GWG*, gestational weight gain; *GA*, gestational age; *HDL-C*, high-density lipoprotein cholesterol; *LDL-C*, low-density lipoprotein cholesterol; *SE*, standard error; *OR*, odds ratio; *95% CI*, lower and upper bounds of 95% confidence interval. A multivariable binary logistic regression model was performed to estimate associations among maternal and neonatal variables and the global neurodevelopmental outcome in infants.

Variables that showed significant differences are indicated in bold.

**Table 3:** Multiple linear regression analysis of BSID-III domain scores.

Domain	Model	Beta	Standardized beta coefficients	95% IC	*p*-value	*R*	Adjusted *R*^2^	*p*-value
Cognitive	Parity	0.89	0.37	(0.22, 1.56)	0.011	0.470	0.182	0.007
	GWG	− 0.06	− 0.28	(− 0.13, − 0.0003)	0.049			
Fine motor	Sex of newborn	− 0.84	− 0.33	(− 1.60, −0.08)	0.032	0.328	0.086	0.032
Socioemotional	Mode of delivery	1.73	0.33	(0.18, 3.27)	0.30	0.332	0.089	0.030

All domains were evaluated with the BSID-III test (cognitive, receptive language, expressive language, fine motor skills, gross motor skills, and socioemotional) using multiple linear regression models. For each domain, the included variables were maternal age, pregestational body mass index (pBMI), parity, glucose, triglyceride, total cholesterol, HDL-C, LDL-C, gestational weight gain (GWG), gestational age at birth (GA), mode of delivery, sex of the newborn, weight, length, and head circumference at birth. Only models and variables with statistically significant associations are presented. *N* = 49 mother–newborn dyads.

In the categorical analysis, the most affected domains were expressive language (40.8%), cognitive (28.6%), and socioemotional (24.5%), as shown in **Table 4**.

**Table 4:** Neurodevelopmental outcomes in infants at 1 month of age.

Domain	Neurodevelopmental outcome	
	Normal *n* (%)	Altered *n* (%)
Cognitive	35 (71.4)	14 (28.6)
Language composite	41 (83.7)	8 (6.30)
Receptive language	46 (93.9)	3 (6.10)
Expressive language	29 (59.2)	20 (40.8)
Motor composite	49 (100)	–
Fine motor	49 (100)	–
Gross motor	48 (98.0)	1 (2.00)
Socioemotional	37 (75.5)	12 (24.5)

Percentages of neurodevelopmental outcomes in infants at 1 month of age. Domains were evaluated using the BSID-III, and an altered neurodevelopment score was defined as a score ≤ 7 (≥ 1 SD below the mean). *N* = 49.

### 3.2 Association of 5-HT levels with infant neurodevelopment outcomes

**Table 5** presents the 5-HT levels measured in maternal serum, umbilical cord serum, and placental tissue. No statistically significant differences were found in 5-TH concentrations between infants with normal *vs.* altered neurodevelopment, either in overall comparisons or domain-specific analyses.

**Table 5:** 5-HT levels in maternal, cord, and placental samples and neurodevelopmental outcomes in infants.

Neurodevelopment outcome	5-TH levels	Normal	Altered	*p*-value
Global neurodevelopment	5-TH_Mat_ (nmol/L)	732 [558–1,074]	846 [557–1,106]	0.689
	5-TH_UC_ (nmol/L)	139 [48.7–478]	197 [87.2–377]	0.890
	5-TH_Pl_ (pg/mg of protein)	0.176 [0.105–0.237]	0.168 [0.125–0.220]	0.876
Cognitive domain	5-TH_Mat_ (nmol/L)	765 [574–1,044]	835 [447–1,150]	0.617
	5-TH_UC_ (nmol/L)	205 [71.8–406]	183 [47.6–402]	0.472
	5-TH_Pl_ (pg/mg of protein)	0.169 [0.107–0.240]	0.164 [0.129–0.210]	0.874
Language composite domain	5-TH_Mat_ (nmol/L)	768 [570–1,150]	578 [488–941]	0.402
	5-TH_UC_ (nmol/L)	197 [85.1–446]	133 [28.5–239]	0.156
	5-TH_Pl_ (pg/mg of protein)	0.169 [0.113–0.216]	0.196 [0.122–0.260]	0.430
Expressive language domain	5-TH_Mat_ (nmol/L)	728 [544–1,116]	910 [565–1,061]	0.507
	5-TH_UC_ (nmol/L)	179 [48.7–426]	186 [93.0–293]	0.973
	5-TH_Pl_ (pg/mg of protein)	0.159 [0.106–0.219]	0.171 [0.142–0.241]	0.229
Socioemotional	5-TH_Mat_ (nmol/L)	768 [544–972]	765 [561–1,292]	0.560
	5-TH_UC_ (nmol/L)	188 [75.2–399]	183 [68.4–494]	0.787
	5-TH_Pl_ (pg/mg of protein)	0.171 [0.124–0.223]	0.147 [0.09–0.245]	0.425

*Mat*, maternal; *UC*, umbilical cord; *Pl*, placental tissue. Continuous variables are expressed as median [interquartile range] and analyzed using the Mann–Whitney *U* test.

### 3.3 Expression of serotonergic system components in placental tissue

**Figure 2:**
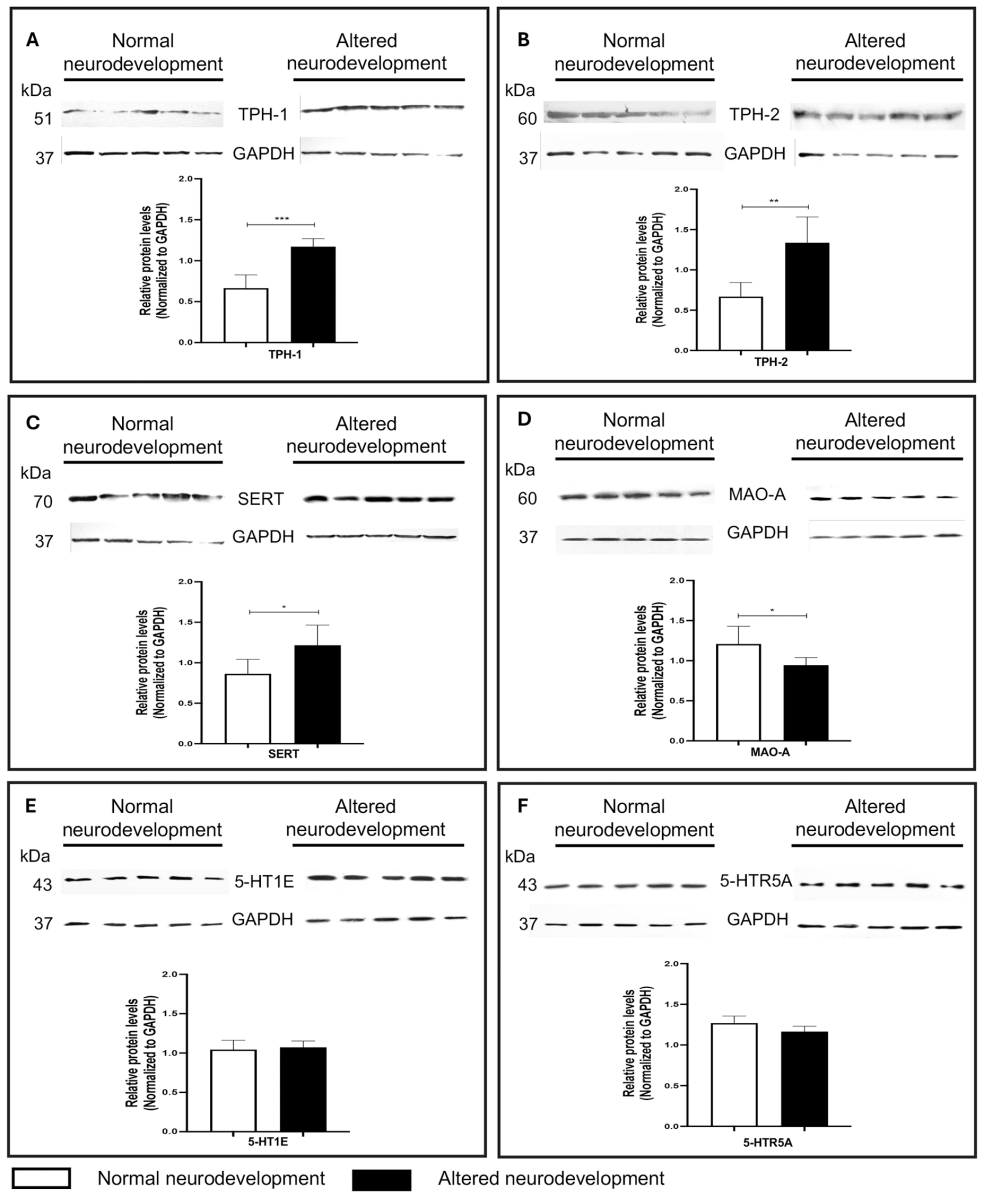
Expression of key serotonergic system proteins in placental tissue from infants with normal and altered neurodevelopment. **(A–F)** Top: representative Western blot images of placental lysates from five infants with normal and five with altered neurodevelopment. Proteins evaluated include the synthesizing enzymes **(A)** TPH1 and **(B)** TPH2, the **(C)** serotonin transporter (SERT), the degrading enzyme **(D)** monoamine oxidase A (MAO-A), and the **(E)** serotonin receptors 5-HT1E and **(F)** 5-HTR5A. Bands were visualized by chemiluminescence and captured on radiographic film. **(A–F)** Bottom: densitometric quantification of TPH1, TPH2, SERT, MAO-A, 5-HT1E, and 5-HTR5A expression, normalized to GAPDH. Data are presented as mean ± SD for each group: normal neurodevelopment (white bars) and altered neurodevelopment (black bars). Comparisons were made using Student’s *t*-test, and results are expressed as mean ± SD. ^*^p<0.05; ^**^p<0.01; ^***^p<0.001.

The expression of TPH1 was 43.21% higher, TPH2 was 50.05%, and SERT was 29.05% higher in placental tissue from infants with altered neurodevelopment compared to infants with normal neurodevelopment. These differences were statistically significant. In contrast, MAO-A levels were 21.77% lower in the altered group. No significant differences were observed in the expression of the 5-HT1E and 5-HTR5A receptors (**Figure 2**).

## 4 Discussion

In this study, we examined placental 5-HT levels and the expression of key proteins of the serotonergic system to explore their association with early infant neurodevelopment. Our findings support the hypothesis that dysregulation of placental serotonergic signaling may be linked to neurodevelopmental impairments, offering new insights into its role in fetal brain development.

5-HT is critical for fetal brain development (30) and participates in numerous neurodevelopmental processes, including neuronal proliferation, migration, apoptosis, morphogenesis, and synaptic connectivity (31), which are influenced by both prenatal and postnatal environmental factors (32). Previous studies have shown that both insufficient and excessive placental production of 5-HT can adversely affect brain development and increase the risk of neurodevelopmental disorders (33, 34). Although we hypothesized that placental 5-HT would differ between infants with normal and altered neurodevelopment, no significant differences were observed. This result contrasts with previous studies that associated abnormal 5-HT levels with neurodevelopmental disorders such as autism spectrum disorder (ASD) and attention-deficit/hyperactivity disorder (ADHD) (33, 34). Notably, many of these studies evaluated circulating 5-HT levels in children postnatally—often years after birth—whereas we focused on 5-HT in placental tissue at birth. This temporal and tissue-specific distinction may account for some of the discrepancies.

The absence of differences in total placental 5-HT suggests that early neurodevelopment may depend more on the regulation of serotonergic pathway components than on absolute 5-HT levels at the end of the pregnancy. Instead, other components of the serotonergic system may play a role, underscoring the complexity of the system’s participation in early brain development (35–37). Our findings highlight significant alterations in the expression of proteins that regulate 5-HT availability and signaling. Specifically, we found that term placentas from infants with altered neurodevelopment exhibited higher levels of TPH1 and TPH2—the rate-limiting enzymes for 5-HT synthesis—and increased expression of the 5-HT transporter (SERT), along with reduced expression of MAO-A, the enzyme responsible for 5-HT degradation.

These alterations suggest a disruption in 5-HT homeostasis, potentially leading to excessive or poorly timed serotonergic signaling during critical periods of fetal brain development, even in the absence of changes in overall 5-HT levels. This finding is consistent with previous evidence showing that the functionality and regulation of components such as TPH1/2, SERT, and MAO-A are central to 5-HT-mediated effects on neurodevelopment (20, 34, 38).

Our results are further supported by studies such as that of Räikkönen et al. (39), who found that higher placental expression of *SLC6A4* (the gene encoding SERT) was associated with behavioral regulation difficulties in neonates, including feeding, crying, and sleeping issues. Similarly, Pehme et al. (20) demonstrated that MAO-A expression in human placentas was positively associated with affect and temperament traits in infants at 1 year of age. Together, these findings underscore the relevance of examining placental serotonergic regulation as a dynamic system with developmental implications.

In addition to the findings on serotonergic system components in the placenta, our results suggest a modest contribution of maternal and neonatal characteristics to neurodevelopment. Maternal cholesterol levels showed a negative association with global neurodevelopment. Variables such as parity, GWG, mode of delivery, and newborn sex were also identified as contributors to BSID-III scores. These results are consistent with previous studies indicating that metabolic and obstetric factors can influence neurodevelopmental trajectories (40–45). The relatively modest effect observed in our study may be due to the homogeneity of the study population.

Several studies have highlighted the importance of 5-HT homeostatic compensation within the fetal–placental–maternal unit, which could also explain our findings. Our study explores the protein levels of some serotonergic system components. However, murine models have demonstrated that the activity of SERT, MAO-A, TPH1, and other serotonergic system components not explored in this study could be simultaneously adapted in the placenta due to the availability of substrates such as tryptophan, the 5-HT itself, or modulators like corticosterone (46). This adaptation could be traced over time until reaching a steady state, as a saturable system must be tightly regulated by the placenta in both murine models and humans. Such regulation is critical due to the significant effects of 5-HT on the circulatory system, including vasoconstriction and edema formation, which can lead to increased blood pressure (46).

A detailed study is needed on the polymorphism status of serotonergic components expressed in placental tissue, which could also underline variations in the activity of the serotonergic system components, rather than differences in 5-HT concentrations per se. This perspective should include a large cohort to explore the potential association of these polymorphisms and an increased risk of adverse neurodevelopmental outcomes (14), alongside evaluating the activity of the proteins involved in 5-HT transport and metabolism.

Importantly, this exploratory study is one of the first in Latin America to report associations between placental serotonergic system dysregulation and infant neurodevelopment, contributing novel data to an area that remains underexplored in low- and middle-income country settings. Our results reinforce the concept of fetal programming, suggesting that disturbances in placental 5-HT signaling may have long-lasting consequences on brain development and mental health across the lifespan.

Numerous genetic and environmental risk factors—including maternal stress, inflammation, nutrition, and environmental exposures—are known to influence placental 5-HT signaling. Future studies should explore the interplay between these factors and the expression or function of placental serotonergic components, as such interactions may contribute to the developmental origins of conditions including ASD, ADHD, and mood disorders.

In conclusion, our study demonstrates that altered expression of serotonergic proteins in the placenta —specifically increased TPH1/TPH2 and SERT, along with reduced MAO-A—could be associated with early neurodevelopment impairments in 1-month-old infants. These findings suggest that the balance and regulation of serotonin signaling during gestation, rather than absolute 5-HT levels, should be key in shaping early brain development. Our results also highlight potential placental biomarkers for identifying infants at risk of neurodevelopmental disorders, which could guide early screening and intervention strategies.

## 5 Limitations of the study

Several limitations in this study should be acknowledged. First, the sample size was relatively small and restricted to participants for whom complete placental tissue, clinical data, and neurodevelopmental follow-up at 1 month were available. This may limit the generalizability of our findings, particularly in relation to the Western blot analyses, which were performed on a subset of only five representative samples per group.

Second, neurodevelopment was assessed using just a single tool—BSID-III. Although this is a widely accepted and standardized instrument, its sensitivity and specificity at very early ages, such as 1 month, are limited. Many developmental domains are still emerging at that age, and assessments often rely on indirect indicators and caregiver input. To address this, we used categorical classification based on standard scores, but the inherent challenges of early infancy assessment remain.

Third, although we quantified serotonergic components in placental tissue, we did not measure these markers in infant serum or other postnatal compartments. Such data could have provided a more comprehensive view of the serotonergic environment to which the developing brain was exposed and its relationship with neurodevelopmental trajectories. We also note that more sensitive methods, such as LC-MS/MS, could be useful for detecting differences in 5-HT levels in serum samples.

Finally, unmeasured confounding factors—such as prenatal maternal stress, environmental exposures, quality of postnatal caregiving, socioeconomic context, and epigenetic regulation—may also influence both placental physiology and infant neurodevelopment. Future studies should integrate multidimensional data to more comprehensively characterize these complex interactions.

Other important considerations are that the high percentage of cesarean sections (C-sections) in our sample reflects two key contextual factors of the population from which participants were recruited. First, among multiparous women, C-sections are often iterative; once a woman has had a previous C-section, subsequent deliveries frequently follow the same route due to clinical recommendations or institutional practices. Second, although the WHO statement on cesarean section rates suggests that only 10%–15% of births should require a C-section (47), Mexico—like many Latin American countries—has persistently high rates of C-section deliveries (48). These elevated rates have been attributed to a combination of clinical, sociocultural, economic, and institutional factors, including routine obstetric practices, provider preference, medicolegal concerns, and patient expectations. Moreover, because our study was conducted within a public health and specialized institution for high-risk pregnancies, where these patterns are well documented, the proportion of C-sections in our cohort likely reflects the local epidemiology of delivery mode rather than a selection bias introduced by our study design. Importantly, C-section was not used as an inclusion criterion, and participants were enrolled independently of their eventual mode of delivery.

## Data Availability

The raw data supporting the conclusions of this article will be made available by the authors, without undue reservation.
